# Prevalence of SARS-CoV-2 infection among people experiencing homelessness in Toronto during the first wave of the COVID-19 pandemic

**DOI:** 10.17269/s41997-021-00591-8

**Published:** 2021-12-17

**Authors:** Linh Luong, Michaela Beder, Rosane Nisenbaum, Aaron Orkin, Jonathan Wong, Cynthia Damba, Ryan Emond, Suvendrini Lena, Vanessa Wright, Mona Loutfy, Cindy Bruce-Barrett, Wilfred Cheung, Yick Kan Cheung, Victoria Williams, Miriam Vanmeurs, Andrew Boozary, Harvey Manning, Joe Hester, Stephen W. Hwang

**Affiliations:** 1 MAP Centre for Urban Health Solutions, Li Ka Shing Knowledge Institute, St Michael’s Hospital, Unity Health Toronto, 30 Bond Street, Toronto, ON M5B 1W8 Canada; 2 Department of Psychiatry, University of Toronto, Toronto, ON Canada; 3 Division of Biostatistics, Dalla Lana School of Public Health, University of Toronto, Toronto, ON Canada; 4 Department of Family and Community Medicine, University of Toronto, Toronto, ON Canada; 5 Inner City Health Associates, Toronto, ON Canada; 6 Central Local Health Integration Network, Ontario Health Toronto, Toronto, ON Canada; 7 Department of Medicine, Women’s College Hospital, Toronto, ON Canada; 8 The Centre for Addiction and Mental Health, Toronto, ON Canada; 9 Department of Family and Community Medicine, Women’s College Hospital, Toronto, ON Canada; 10 Women’s College Research Institute, Women’s College Hospital, University of Toronto & Maple Leaf Medical Clinic, Toronto, ON Canada; 11 The Hospital for Sick Children, Toronto, ON Canada; 12 University Health Network, Toronto, ON Canada; 13 Anishnawbe Health Toronto, Toronto, ON Canada

**Keywords:** Homelessness, COVID-19 pandemic, Homeless shelters, Vulnerable populations, Sans-abrisme, pandémie de COVID-19, refuges pour sans-abri, populations vulnérables

## Abstract

**Objectives:**

People experiencing homelessness are at increased risk of SARS-CoV-2 infection. This study reports the point prevalence of SARS-CoV-2 infection during testing conducted at sites serving people experiencing homelessness in Toronto during the first wave of the COVID-19 pandemic. We also explored the association between site characteristics and prevalence rates.

**Methods:**

The study included individuals who were staying at shelters, encampments, COVID-19 physical distancing sites, and drop-in and respite sites and completed outreach-based testing for SARS-CoV-2 during the period April 17 to July 31, 2020. We examined test positivity rates over time and compared them to rates in the general population of Toronto. Negative binomial regression was used to examine the relationship between each shelter-level characteristic and SARS-CoV-2 positivity rates. We also compared the rates across 3 time periods (T1: April 17–April 25; T2: April 26–May 23; T3: May 24–June 25).

**Results:**

The overall prevalence of SARS-CoV-2 infection was 8.5% (394/4657). Site-specific rates showed great heterogeneity with infection rates ranging from 0% to 70.6%. Compared to T1, positivity rates were 0.21 times lower (95% CI: 0.06–0.75) during T2 and 0.14 times lower (95% CI: 0.04–0.44) during T3. Most cases were detected during outbreak testing (384/394 [97.5%]) rather than active case finding.

**Conclusion:**

During the first wave of the pandemic, rates of SARS-CoV-2 infection at sites for people experiencing homelessness in Toronto varied significantly over time. The observation of lower rates at certain sites may be attributable to overall time trends, expansion of outreach-based testing to include sites without known outbreaks, and/or individual site characteristics.

**Supplementary Information:**

The online version contains supplementary material available at 10.17269/s41997-021-00591-8.

## Résumé

**Objectifs:**

Les personnes en situation d’itinérance courent un risque accru de contracter une infection par le SRAS-CoV-2. Notre étude rend compte de la prévalence ponctuelle des infections par le SRAS-CoV-2 au cours de tests de dépistage menés dans des lieux de services aux personnes en situation d’itinérance de Toronto au cours de la première vague de la pandémie de COVID-19. Nous avons aussi exploré l’association entre les caractéristiques de ces lieux et les taux de prévalence.

**Méthode:**

L’étude a inclus les personnes séjournant dans des refuges, des campements, des lieux de distanciation physique et des centres d’accueil et de répit et ayant subi un test de dépistage de proximité du SRAS-CoV-2 entre le 17 avril et le 31 juillet 2020. Nous avons examiné les taux de positivité des tests au fil du temps et nous les avons comparés aux taux dans la population générale de Toronto. Des analyses de régression binomiales négatives ont été effectuées pour étudier la relation entre chaque caractéristique au niveau des refuges et les taux de positivité au SRAS-CoV-2. Nous avons aussi comparé les taux de trois intervalles (I1: 17 au 25 avril; I2: 26 avril au 23 mai; I3: 24 mai au 25 juin).

**Résultats:**

La prévalence globale des infections par le SRAS-CoV-2 était de 8,5 % (394/4 657). Les taux d’infection spécifiques aux lieux de services ont présenté une grande hétérogénéité, soit de 0 % à 70,6 %. Comparés au 1^er^ intervalle (I1), les taux de positivité ont été 0,21 fois plus faibles (IC de 95% : 0,06 – 0,75) durant l’I2 et 0,14 fois plus faibles (IC de 95% : 0,04 – 0,44) durant l’I3. La plupart des cas ont été détectés lors d’un dépistage en cours d’éclosion (384/394 [97,5%]) et non lors d’une recherche active de cas.

**Conclusion:**

Au cours de la première vague de la pandémie, les taux d’infection par le SRAS-CoV-2 dans les lieux de services aux personnes en situation d’itinérance de Toronto ont varié de façon significative au fil du temps. L’observation de taux plus faibles dans certains lieux pourrait s’expliquer par les tendances temporelles globales, par l’expansion des activités de dépistage de proximité pour inclure les lieux sans éclosion connue et/ou par les caractéristiques individuelles des lieux.

## Introduction

The COVID-19 pandemic has disproportionately impacted and continued to pose extraordinary challenges for people experiencing homelessness (Perri et al., 2020; Turnbull et al., 2021), especially those from Indigenous and racialized communities. Prior to the pandemic, many of these individuals were already struggling due to low social assistance rates, the opiate crisis, and a chronic lack of sufficient shelter beds. More than 35,000 people experience homelessness on any given night in Canada, and at least 235,000 individuals were homeless in a year (Gaetz et al., 2016). The homeless populations in Toronto are quite diverse; Indigenous and racial/ethnic minority groups are overrepresented, constituting about 10% and 54%, respectively, of homeless population in Toronto (City of Toronto, 2018). In particular, people experiencing homelessness who identified as Indigenous are overrepresented in the outdoor homeless populations (36%). There is also a great contrast between outdoor and indoor homeless populations, with men experiencing homelessness more likely to be sleeping outdoors (73%) compared to women (City of Toronto, 2018). Furthermore, the majority of individuals staying at single adults (67%) and 24-hour respite sites (59%) are male, whereas women are overrepresented in family shelters (75%). A total of 29% of people experiencing homelessness reported living both outdoors and in shelters in 2018 (City of Toronto, 2018). Moreover, youth (16 to 24 years old) represent 10% of the Toronto homeless population. There is also an increasing number of refugees and asylum claimants (40%) in the Toronto shelter system in 2018 compared to previous years, and 80% of those living in the family shelters identified themselves as refugees/asylum claimants (City of Toronto, 2018).

People experiencing homelessness are at increased risk for infectious diseases because of their poor health and complex health concerns ranging from physical to mental illnesses and addiction and substance-use issues (Hwang, 2001). In addition, crowding and shared living spaces in shelters make it difficult for residents to adhere to pandemic-related physical distancing guidelines. Furthermore, the number of homeless persons living in outdoor encampments has risen during the pandemic, raising questions about whether the risk for SARS-CoV-2 infection in encampments is higher or lower than in shelters. Earlier studies of SARS-CoV-2 infection rates across various outbreak settings in the Greater Toronto Area have found a sharp shift from travel-related and community-related cases to congregate-related cases, especially in long-term care (LTC) homes between January 23 and May 20, 2020 (Wang et al., 2020). In particular, the epidemic curve of LTC homes peaked in early April. In response to the increase in the number of outbreaks in congregate settings, the Government of Ontario had expanded the testing criteria to include asymptomatic individuals who were in close contact with positive cases in LTC, shelters, etc. (Government of Ontario, 2020a).

Few studies have examined SARS-CoV-2 infection in people experiencing homelessness in both shelters and encampments in Canada. Therefore, the objective of this study was to estimate the prevalence of SARS-CoV-2 infection among people experiencing homelessness in the City of Toronto who received outreach-based testing at shelters, drop-ins, and encampments during the first COVID-19 pandemic wave. We also explored the association between site characteristics and prevalence rates.

## Methods

### Study design and setting

This study is a retrospective review of aggregated data collected by Ontario Health Toronto as part of outreach testing for SARS-CoV-2 infection among individuals experiencing homelessness in Toronto. This was a combination of two testing programs: (1) an active case-finding program involving testing in shelters without identified cases throughout the study period, and (2) an outbreak management program involving testing in response to positive cases that were identified through community testing or symptoms. Testing was conducted across various types of sites, including shelter-based programs, drop-in and respite programs, encampments, and COVID-19 physical distancing sites, which are sites that were opened during the pandemic to allow safe physical distancing for shelter residents. The study included data on testing performed on residents at these sites between April 17 and July 31, 2020, which included the first wave of Ontario’s COVID-19 pandemic. Each on-site testing was done either for active case finding or for outbreak management purposes. Outbreak was defined as having at least one laboratory-confirmed case in residents or staff. This is in accordance with the Ontario Ministry of Health’s outbreak guidance for congregate settings (Ministry of Health & Long-Term Care, 2020). Sites not indicated for outbreak testing were classified as active case finding.

### Participants

Shelters, drop-ins and respite sites, encampments, and COVID-19 physical distancing sites that completed nasopharyngeal swab tests for SARS-CoV-2 infection between April 17 and July 31, 2020 were eligible to be included in our study. We excluded testing at boarding homes, transitional housing, and permanent housing. We also excluded dedicated COVID-19 isolation and recovery sites as all individuals at these sites were either confirmed positive, presenting with COVID-19 symptomology, or were close contacts with a positive case prior to their admission to the sites. As many sites were tested more than once during the study, we only included results from the first testing period for each site to reduce double counting of individuals tested.

### Data sources

We used aggregated data from Ontario Health Toronto, Toronto Public Health (TPH), and the City of Toronto Shelter, Support and Housing Administration (SSHA) to ascertain outbreak status, COVID-19 test results, and shelter characteristics. Data from Ontario Health Toronto contained information on test dates, total number of residents tested, the number tested positive for each date, and the location of each site. The outreach shelter-based testing was conducted by various community agencies and healthcare organizations. TPH data were used to determine outbreak status for each testing date. We also used two separate TPH datasets to ascertain weekly laboratory positivity rates (per 100 individuals tested) of the Toronto general population, and the number of sporadic cases by neighbourhood regions (City of Toronto, 2020). The weekly positivity rate in the general population in Toronto was defined as the number of people who had a COVID-19-positive test result per 100 people tested each week (Sunday to Saturday) (City of Toronto, 2020). Sporadic cases were defined as those occurring in the community that are not associated with outbreaks in healthcare and congregate settings (e.g., shelters). Sporadic cases were reported by Toronto neighbourhoods and by reported date (e.g., date that a person acquired the disease). At the time of our study, the data included all sporadic cases as early as January 21 to July 31, 2020*.* We collected shelter characteristics such as layout style and population type of each site using data from SSHA.

### Primary outcome

The primary outcome was the prevalence of SARS-CoV-2 infection at each site between April 17 and July 31, 2020. The prevalence was defined as the number of confirmed positive cases divided by total number of residents tested on each date. Sites with a large number of residents were tested over two or more days.

### Site characteristics

Sites were grouped into four mutually exclusive categories: shelter, drop-in/respite, encampment, and COVID-19 physical distancing site. Shelter population was categorized by groups (youth, single adults, and families) and gender (men, women and mixed). We further categorized site type as to whether or not it served exclusively refugee claimants. For each site, we defined its layout as one of the following: single room, shared room (2–5 beds), dorm or open style (more than 6 beds), and encampment (outdoor). If a site had a mixed layout (both single rooms and shared rooms), we classified the layout as shared rooms to reflect COVD-19 transmission risk in shared spaces.

As a proxy measure of COVID-19 transmission in the community that could potentially affect site positivity rates, data were obtained on the number of sporadic cases of COVID-19 (i.e., cases not associated with outbreaks in healthcare and congregate settings) in the neighbourhood where the sites were located.

### Statistical analyses

Prevalence of SARS-CoV-2 infection was expressed as positivity rates per 100 tested residents between April 17 and July 31, 2020, and it is described by site and by site characteristics. The overall prevalence of SARS-CoV-2 infection was calculated by dividing the total number of confirmed cases across all sites by the total number of tested individuals during the study period with 95% confidence interval. We also compared the weekly COVID-19 positivity rates among individuals screened at sites for people experiencing homelessness in relationship to the positivity rates in the general Toronto population.

The association between site characteristics and site prevalence rates was evaluated using a negative binomial regression. The model’s outcome was the total number of positive cases in each site and included one site characteristic and the log of the total number of residents tested as the offset. Only univariate models were fit, since most the site characteristics were highly correlated. For our regression analysis, we focused on data between April 17 and June 25, 2020, since this period constituted most of the first wave of the pandemic. In addition, on June 25, 2020, Toronto entered stage 2 wherein businesses and services were allowed to reopen (Government of Ontario, 2020b). We categorized our testing dates into 3 periods: T1, April 17–April 25; T2, April 26–May 23; T3, May 24–June 25, 2020. Rate ratios (RR) and 95% confidence interval (CI) estimated ratios of daily prevalence rates. We hypothesized that new outbreaks in shelter sites may be associated with the accumulation of SARS-CoV-2 transmission in the community within the neighbourhood where each site is located. To compare outbreaks in homeless sites with sporadic cases in community, for each site, we calculated the cumulative sporadic cases from January 21, 2020 (when the data were first available) up until the date of the testing by Toronto neighbourhood regions. All analyses were performed using SAS 9.4 (SAS Institute Inc., Cary, NC, USA).

## Results

The final sample included 97 unique sites and 111 testing dates. Eight sites had to perform testing on multiple days because of high resident volume, which led to purposefully testing over more than 1 day, or because not all residents were available on the first day of testing. Among the excluded testing dates (*n* = 49), 8 had missing test results, 23 were not at a site for people experiencing homelessness, and 18 were repeated testing.

Table 1 presents characteristics of the sites and testing completed between April 17 and July 31, 2020. The majority were shelter sites (58.8%). Most sites had a shared room layout (43.8%). At 92% of sites, testing was performed on only a single date. Testing was performed in response to an outbreak on 26.8% of the dates and for active case-finding purposes (in the absence of any known outbreak) in 73.2% of the dates. In particular, between April 17 and June 25, there were 69 testing dates, and 58.0% of them were for outbreak purposes. After June 25, all 42 testing dates were performed for active case-finding purposes.

**Table 1:** Testing characteristics of study sites between April 17 and July 31, 2020

Description	*N* (%)
Number of sites	97
Sector	
Shelter	57 (58.8)
Drop-in and respite programs	12 (12.4)
Encampment site	9 (9.3)
COVID-19 response sites and hotels	19 (19.6)
Population group	
Youth	8 (8.3)
Single adults	79 (81.4)
Families	10 (10.3)
Population gender	
Men	25 (25.8)
Women	22 (22.7)
Mixed	50 (51.6)
Refugee sites	7 (7.2)
Reason for testing	
Outbreak	26 (26.8)
Active case finding	71 (73.2)
Layout^†^	
Single room	20 (20.8)
Shared room	42 (43.8)
Dorm style or open layout	25 (26.0)
Encampment	9 (9.4)
Number of testings conducted by time period (*n* = 111)	
17 Apr–25 Apr	8 (7.2)
26 Apr–23 May	19 (17.1)
24 May–20 Jun	31 (27.9)
21 Jun–18 Jul	42 (37.8)
19 Jul–31 Jul	11 (9.91)

^†^Missing for one site

**Fig. 1:**
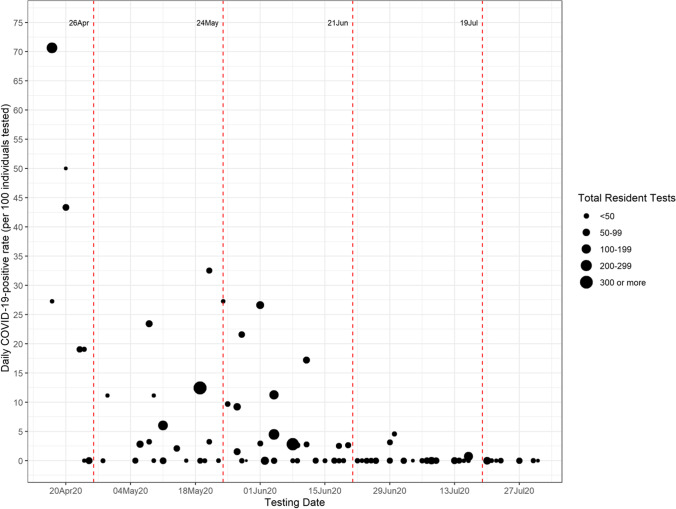
Daily positivity rates from April 1 to July 31, 2020. The size of the circles represents the number of residents tested on each day

**Fig. 2:**
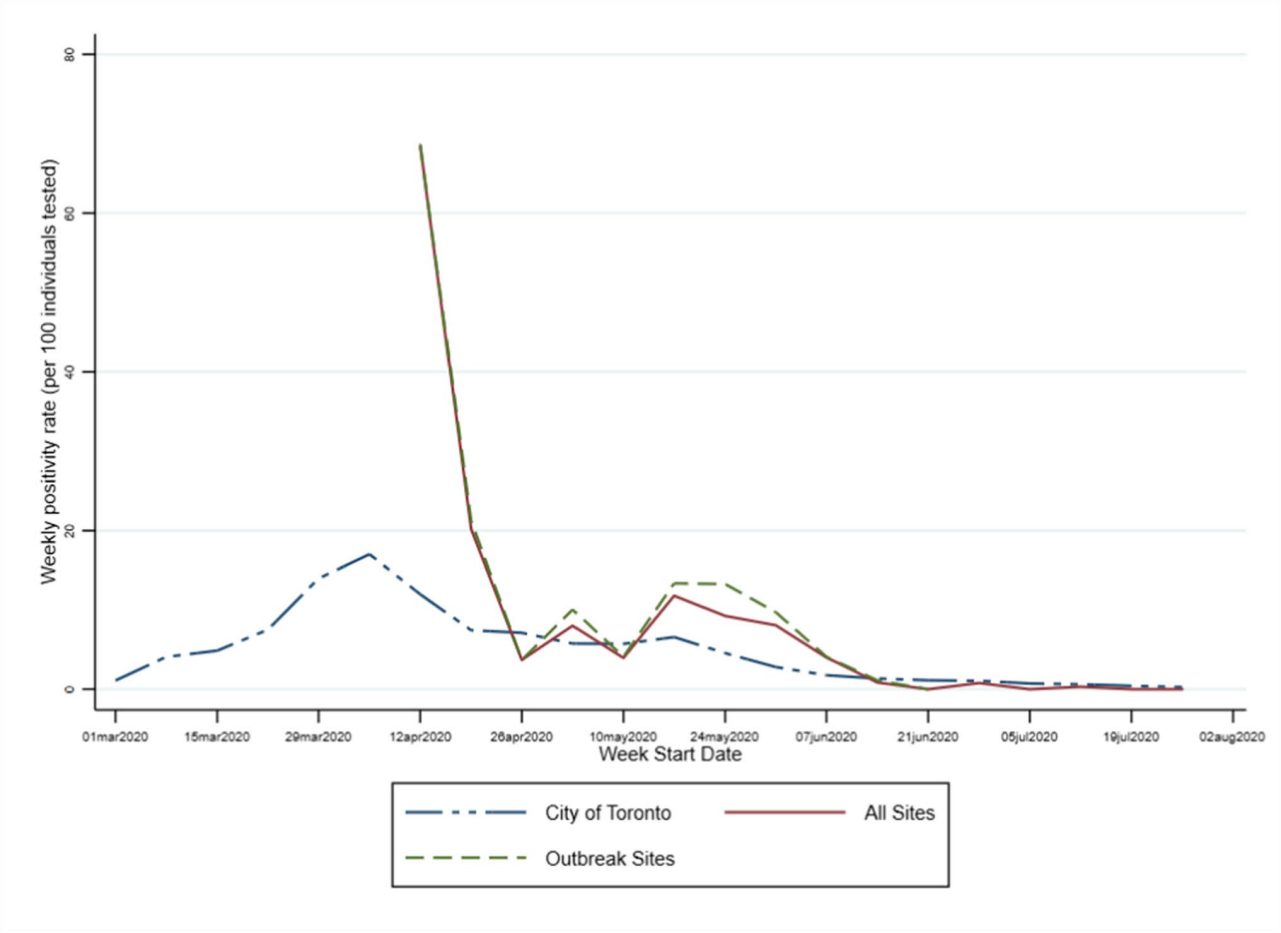
Weekly COVID-19 positivity rates (per 100 individuals tested) for the weeks starting on March 1, 2020 and ending on the week starting on July 26, 2020

The overall prevalence of SARS-CoV-2 infection among shelter residents between April 17 and July 31, 2020, was 8.5% (394/4657, 95% CI 7.7–9.3%). For the period of April 17 to June 25, the prevalence was 11.5% (391/3415, 95% CI 10.4–12.5%). Site-specific rates ranged from 0 to 70.6% and declined over time (Fig. 1). After June 20, the prevalence was essentially zero. Most cases were detected during outbreak testing (384/394 [97.5%]) rather than active case finding. Compared to the City of Toronto epidemic curve, the Toronto shelter positivity rates were consistently higher, with the exception of the period between April 26 and May 10 (Fig. 2). All youth shelters were tested after May 24, 2020, and their SARS-CoV-2 infection rates were essentially zero. Given little variability in the youth shelters sample for comparison, we combined them with family shelters in our regression analysis.

The daily prevalence rates of SARS-CoV-2 during T2 and T3 were significantly lower compared to the rates during T1 (Table 2). Compared with T1, T2 and T3 had 79% (RR: 0.21, 95% CI: 0.06–0.75) and 86% (RR: 0.14, 95% CI: 0.04–0.44) lower rates, respectively. We observed significantly lower rates in drop-in/respite program (RR: 0.23, 95% CI: 0.07–0.80) and encampment sites (RR: 0.05, 95% CI: 0.004–0.62) compared to shelters. The prevalence rate at encampments was significantly lower (RR: 0.04, 95% CI: 0.002–0.63) compared to sites with a single room layout. Refugee sites showed a higher prevalence of SARS-CoV-2 compared to non-refugee sites, but the association was not significant (RR: 3.15, 95% CI: 0.68–14.6). The number of sporadic cases of COVID-19 in the community where the site was located was not associated with site-confirmed positive cases. Given there was an outlier data point with 70.6% positivity rate, we conducted a sensitivity analysis excluding this site. The model results were virtually the same, except that the positivity rate of period T2 was no longer significantly lower compared to period T1 (Online Resource 1).

**Table 2:** Association between each site characteristic and the number of daily confirmed COVID-19 cases (*n* = 69)

	Rate ratios (RR)	95% CI	*p*-value
Time period			
T1	1.00	–	–
T2	0.21	0.06, 0.75	0.016
T3	0.14	0.04, 0.44	0.001
Shelter sector			
Shelter	1.00	–	–
Drop-in/respite program	0.23	0.07, 0.80	0.021
Encampment	0.05	0.004, 0.62	0.020
COVID physical distancing sites	1.12	0.34, 3.71	0.850
Shelter layout			
Single room	1.00	–	–
Shared room	0.64	0.17, 2.48	0.518
Open layout or dorm style	0.66	0.17, 2.51	0.540
Encampment (outdoor)	0.04	0.002, 0.63	0.022
Shelter group by age			
Families/youth	1.00	–	–
Single adult	0.44	0.13, 1.50	0.19
Shelter group by gender			
Men	1.0	–	–
Women	1.21	0.32, 4.67	0.779
Mixed	0.99	0.36, 2.71	0.980
Community transmission^a^	0.97	0.90, 1.04	0.365
Refugee site	3.15	0.68, 14.6	0.143

Rate ratios and 95% confidence intervals estimated by fitting univariate negative binomial models including the site characteristics and log of total number of tests as the offset

^a^ For every increase of 10 community cases

## Discussion

The study found great heterogeneity (0–70.4%) in the prevalence of SARS-CoV-2 infection across shelters in Toronto during the period April 17 to July 31, 2020. We found significantly higher positivity rates during the T1 (April 17–April 25) compared to later testing periods. We also found that site-based active case finding (testing in the absence of an outbreak) identified few cases and likely provided minimal benefits in terms of reducing the transmission of SARS-CoV-2. This finding is consistent with modeling studies that indicated that mass testing has to occur every 7 days to be a useful part of shelter COVID management (Baggett et al., 2020). Positivity rates were significantly lower at drop-in/respite programs and encampment sites than at shelter sites.

This study provides insights into SARS-CoV-2 infection rates and shelter-level factors associated with positivity rates in the homeless population in Toronto. Our prevalence (8.5%) is higher compared to previous Ontario-based studies that used administrative health records to ascertain positivity rates (2.3–6.4%) in homeless populations (Richard et al., 2021; Wang et al., 2020). Since our study sample was drawn from sites serving people experiencing homelessness rather than users of the healthcare system who were homeless, our estimate may better reflect the positivity rate among people experiencing homelessness. Our rate is lower compared to prior studies conducted in shelters experiencing outbreaks, which found rates ranging from 18.0% to 41.7% (Baggett, et al., 2020; Redditt et al., 2020; Tobolowsky et al., 2020). In contrast, we included active case-finding and outbreak testing, which likely contributed to the observation of lower positivity rates.

The rapid decline in positivity rates over time may be attributed to the efficient coordination among shelter providers, TPH, and hospital partners to screen and isolate infected residents at a COVID-19 recovery site, thus reducing the transmission of SARS-CoV-2. There is growing evidence that poor ventilation in enclosed indoor spaces may increase risk of COVID-19 transmission (Bhagat et al., 2020), and this may explain why we observed lower positivity rates in encampments. However, given the small sample size of encampment residents, this finding should be interpreted with caution. The lower positivity rates observed in drop-in and respite programs may be due to the shorter amount of time that people experiencing homelessness spent at these sites compared to shelter sites. The study has limitations. We used aggregate data at the site level, and thus, individual-level characteristics such as symptoms, age, and other sociodemographic characteristics could not be examined. There is a possibility of sampling bias, as sites chosen for testing were based on identification of positive cases or at the request of shelter providers. In addition, at some sites, the proportion of eligible residents who agreed to be tested was low. Given our sample size, we did not control for potential confounders in our models.

## Conclusion

Our study found a high prevalence of SARS-CoV-2 infection among people experiencing homelessness in Toronto during the first wave of the COVID-19 pandemic. This highlights the urgent need to implement measures to end homelessness, including by creating more affordable and supportive housing, increasing social assistance rates, and providing ongoing health and social support to individuals and communities most impacted by COVID, as well as implementing interim measures to decrease COVID transmission risk, such as providing sufficient shelter space to allow for physical distancing, secure employment and paid sick days for staff, and sufficient PPE. Future work should examine the effectiveness of relocation of residents to COVID-19 response hotels/sites and other safety measures to ensure appropriate physical distancing in shelters in reducing COVID-19 transmission.

## **Contributions to knowledge**

What does this study add to existing knowledge?

- This study provides insights into SARS-CoV-2 positivity rates and the site characteristics associated with an increase in positivity rates in homeless populations in Toronto, Canada.

What are the key implications for public health interventions, practice or policy?

- Our findings highlight the urgent need to implement timely and coordinated crisis management to prevent and mitigate the negative consequences of the COVID-19 pandemic in homeless populations and those who serve them.
- Long-term strategies need to be in place to protect the homeless community in the event of a similar crisis in the future by creating more affordable and supportive housing, providing sufficient shelter space to avoid overcrowding, offering social assistance supports, and developing evidence-informed outbreak and infection control guidelines.

## Supplementary Information

Below is the link to the electronic supplementary material.

Supplementary file1 (DOCX 17 KB)

## Data Availability

Not applicable.
