# Supplementary material for: Prevalence of SARS-CoV-2 infection among people experiencing homelessness in Toronto during the first wave of the COVID-19 pandemic
Source: Can J Public Health. 2021 Dec 17;113(1):117–25. doi: 10.17269/s41997-021-00591-8 (PMC8678973; doi:10.17269/s41997-021-00591-8)
Supplement: Supplementary file 1 — Supplementary file1 (DOCX 17 KB) [file 41997_2021_591_MOESM1_ESM.docx]

**Supplemental file**

**Title**: Prevalence of SARS-CoV-2 infection among people experiencing homelessness in Toronto during the first wave of the COVID-19 pandemic

Canadian Journal of Public Health

Linh Luong^1^, Michaela Beder^2^, Rosane Nisenbaum^1,3^, Aaron Orkin^4^, Jonathan Wong^5^, Cynthia Damba^6^, Ryan Emond^6^, Suvendrini Lena^7,8^, Vanessa Wright^9^, Mona Loutfy^10^, Cindy Bruce-Barrett^11^, Wilfred Cheung^6^, Yick Kan Cheung^6^, Victoria Williams^6^, Miriam Vanmeurs^6^, Andrew Boozary^12^, Harvey Manning^13^, Joe Hester^13^, Stephen W. Hwang^1^

^1^ MAP Centre for Urban Health Solutions, Li Ka Shing Knowledge Institute, St Michael’s Hospital, Unity Health Toronto, 30 Bonds Street, Toronto, ON M5B 1W8, Canada

^2^ Department of Psychiatry, University of Toronto, Toronto, Ontario, Canada

^3^ Division of Biostatistics, Dalla Lana School of Public Health, University of Toronto, Toronto, Ontario, Canada

^4^ Department of Family and Community Medicine, University of Toronto, Toronto, Ontario, Canada

^5^ Inner City Health Associates, Toronto, Ontario, Canada

^6^ Central Local Health Integration Network, Ontario Health Toronto, Canada

^7^ Department of Medicine, Women’s College Hospital, Toronto, Ontario, Canada

^8^ Centre for Addiction & Mental Health, Toronto, Ontario, Canada

^9^ Department of Family and Community Medicine, Women’s College Hospital, Toronto, Ontario, Canada

^10^ Women's College Research Institute, Women's College Hospital, University of Toronto & Maple Leaf Medical Clinic, Toronto, Ontario, Canada

^11^ The Hospital for Sick Children, 180 Dundas Street West, Toronto, Ontario, M5G 1Z8

^12^ University Health Network, Toronto, Ontario, Canada

^13^Anishnawbe Health Toronto, Toronto, Ontario, Canada

**Corresponding author:**

Dr. Stephen W. Hwang

MAP Centre for Urban Health Solutions, Li Ka Shing Knowledge Institute, St. Michael's Hospital, 30 Bond St, Toronto, ON, M5B 1W8, Canada

Email: [Stephen.Hwang@unityhealth.to](mailto:Stephen.Hwang@unityhealth.to)

Tel: 416-864-6060 Ext. 5991

Online Resource 1: Association between each site characteristics and the number of daily confirmed COVID-19 cases, excluding the outlier site (n=68)

|  | **Rate ratios (RR)** | **95 % CI** | **p-value** |
| --- | --- | --- | --- |
| Time period |  |  |  |
| *T1* | 1.00 | -- | -- |
| *T2* | 0.27 | 0.07, 1.08 | 0.064 |
| *T3* | 0.18 | 0.05, 0.63 | 0.008 |
| Shelter sector |  |  |  |
| Shelter | 1.00 |  |  |
| Drop-in/respite program | 0.29 | 0.09, 0.95 | 0.042 |
| Encampment | 0.06 | 0.0050, 0.73 | 0.0272 |
| COVID physical distancing sites | 1.3647 | 0.4315, 4.3163 | 0.5966 |
| Shelter layout |  |  |  |
| Single room | 1.00 | -- | -- |
| Shared room | 0.6516 | 0.1830, 2.3202 | 0.5086 |
| Open layout or dorm style | 0.4259 | 0.1183, 1.5329 | 0.1915 |
| Encampment (outdoor) | 0.0388 | 0.0026, 0.5809 | 0.0186 |
| Shelter group by age |  |  |  |
| Families/youth | 1.00 | -- | -- |
| Single adult | 0.98 | 0.27, 3.59 | 0.971 |
| Shelter group by gender |  |  |  |
| Men | 1.00 | -- | -- |
| Women | 1.20 | 0.34, 4.27 | 0.780 |
| Mixed | 0.69 | 0.26, 1.81 | 0.449 |
| Community transmission^a^ | 0.98 | 0.92, 1.05 | 0.578 |
| Refugee site | N/a | N/a | N/a |

^a^ For every increase of 10 community cases

Relative rate ratio for refugee site could not be calculated due to small sample size
